# Invasive Streptococcus Pneumoniae Septicemia Complicated with Hemolytic Uremic Syndrome and Meningitis

**DOI:** 10.7759/cureus.10644

**Published:** 2020-09-25

**Authors:** Vijayakumary Thadchanamoorthy, Kavinda Dayasiri

**Affiliations:** 1 Clinical Sciences Department, Faculty of Health Care Sciences, Eastern University, Batticaloa, LKA; 2 Internal Medicine: Pediatrics, Base Hospital, Mahaoya, LKA

**Keywords:** pneumococcal meningitis, hemolytic uremic syndrome

## Abstract

Streptococcus pneumoniae-associated hemolytic uremic syndrome (SpHUS) is an uncommon cause of hemolytic uremic syndrome (HUS). The diagnosis and treatment of Streptococcus pneumoniae-associated HUS is often difficult and associated with high long-term morbidity and mortality. The authors report a five-year-old child who developed HUS following an invasive Streptococcus (S.) pneumoniae infection. The child initially presented with fever, cough, and difficulty in breathing for three days duration and was clinically and radiologically diagnosed as having right middle lobe pneumonia. Blood culture grew Streptococcus pneumoniae. The cerebrospinal fluid analysis also showed Streptococcus pneumoniae. He was initially treated with intravenous cefotaxime. As the child had a poor response to cefotaxime with ongoing fever, antibiotics were changed to ceftriaxone and vancomycin. Although fever started to subside subsequently, the child deteriorated with reduced urine output and developed generalized body swelling. The hematological and biochemical evaluation confirmed hemolytic uremic syndrome. He needed continuous renal replacement therapy for five days and antibiotics were given for 14 days. He had no long-term sequelae on follow-up.

## Introduction

Hemolytic uremic syndrome (HUS) is a triad of microangiopathic hemolytic anemia, thrombocytopenia, and acute kidney injury. HUS is the most common cause of acute renal failure in children. It is mostly caused by Shiga-like toxins [1-3], and the etiology for Shiga-like toxin-associated HUS is usually enterohaemorrhagic Escherichia coli (E. coli) 0157:H7. However, HUS can be caused by other infections such as non 0157:H7 E. coli serotypes, Shigella dysenteriae, Aeromonas species, human immunodeficiency virus (HIV), and neuraminidase-producing organisms such as influenza A virus and Streptococcus (S.) pneumoniae [4-6]. Hereditary, drug-induced, disease-associated and idiopathic HUS have also been identified [3]. Streptococcus pneumoniae is an uncommon cause of HUS. The mortality and long-term morbidity rates of Streptococcus pneumoniae-associated HUS (SpHUS) are higher than Escherichia coli-associated HUS. With the introduction of a multivalent pneumococcal vaccine (Prevnar 13), a change in the epidemiology of SpHUS is expected soon in many countries [7]. We report a child who developed a complicated course following invasive Streptococcus pneumoniae infection leading right middle lobe pneumonia and meningitis, subsequently, further complicated with HUS needing short term renal replacement therapy.

## Case presentation

A five-year-old boy presented with fever, cough, and difficulty in breathing for three days. He also had right-sided abdominal pain and post-tussive vomiting for two days. He had no history of travel or food from outside. Past medical history was unremarkable apart from having bronchial asthma since the age of two years. After he was first seen by the general practitioner and significant leukocytosis (41x 103/cumm) was noted, he was referred to the tertiary care hospital for further management.

The initial assessment at the tertiary hospital revealed that he was febrile (40 C), ill, irritable, and dyspnoeic. His vital parameters were: respiratory rate - 50/minute, pulse rate - 110 beats /minute, and blood pressure - 90/60 mmHg. His oxygen saturation had been maintained within normal limits whilst oxygen via nasal prongs was administered at 2 liters/minute. Chest examination revealed dullness to percussion and decreased breath sounds in the right middle zone with widespread rhonchi heard all over the chest. Other systems examination was clinically normal on admission.

**Figure 1:**
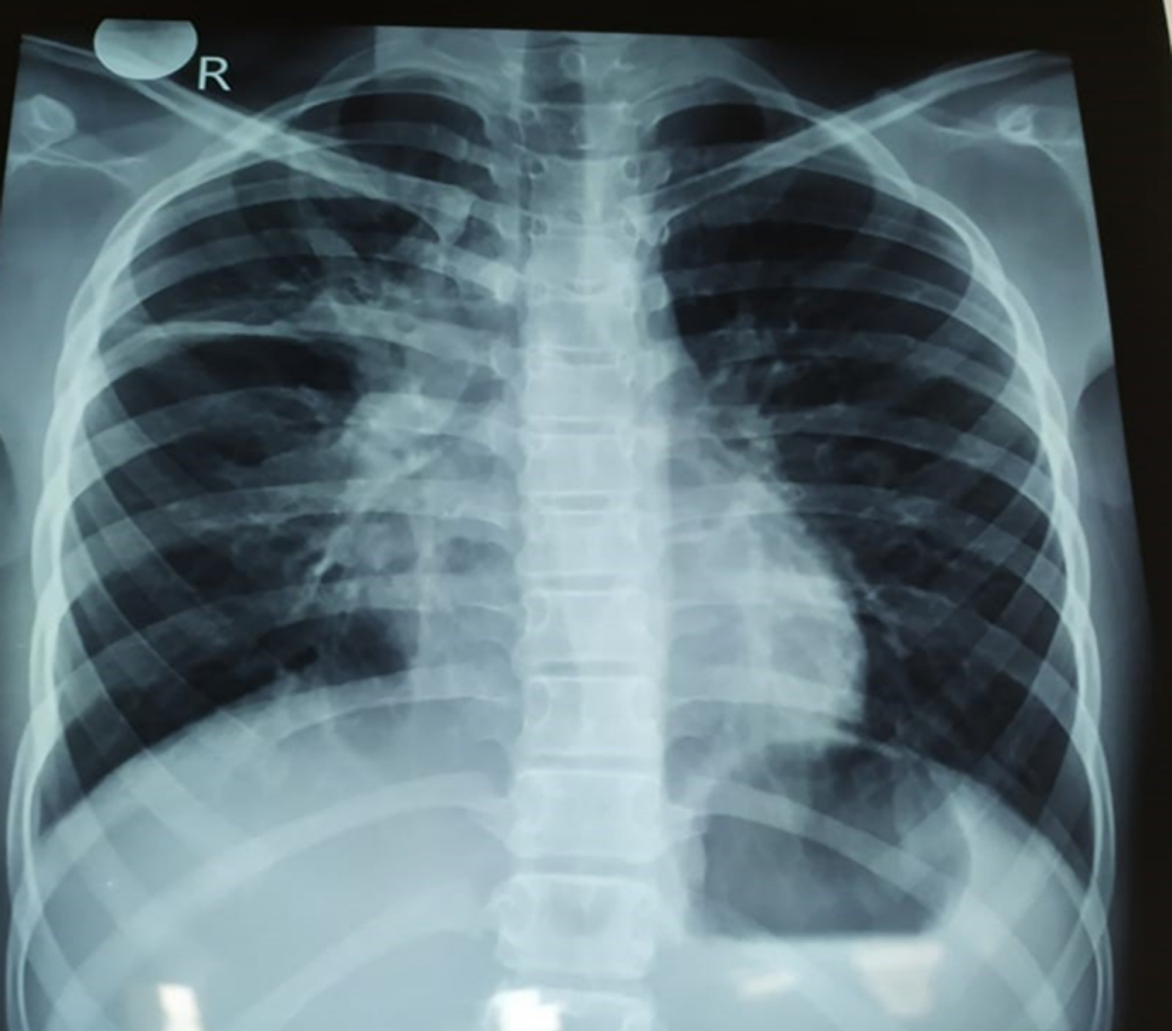
Chest X-ray showing right middle lobe consolidation

Investigations revealed leukocytosis with predominant neutrophilia (white cells - 38x103/cumm, neutrophils - 91%, Hb - 12g/dl, platelets - 225x10/mm), and high C-reactive protein (234 mg/dl). Chest X-ray (CXR) confirmed right middle lobe consolidation (Figure 1). His renal function on admission was normal. He was commenced on intravenous cefotaxime along with supportive treatment. As he had poor response following 72 hours of treatment with intravenous (IV) cefotaxime, a lumbar puncture was performed. The cerebrospinal fluid analysis showed significant polymorphs (90/cumm) and high protein (120 mg/dl). Blood culture grew Streptococcus pneumoniae. The antibiotic was changed subsequently to ceftriaxone and vancomycin according to the sensitivity pattern prevailing presently in this region and later being agreed with antibiotic sensitivity pattern in blood culture. Although his fever responded to change in antibiotics, he continued to be ill and developed reduced urine output and mild generalized body swelling on day five of admission. Re-examination showed pallor, significant periorbital swelling, tachycardia (more than 150 bpm), and high blood pressure (150/80 mmHg). Repeat investigations showed persistently high leukocytes (32x103, N - 74%), acute onset anaemia (Hb - 7 g/dl), and thrombocytopenia (platelets - 94x103/cumm). Inflammatory markers remained elevated (C-reactive protein (CRP) - 124 mg/dl, erythrocyte sedimentation rate (ESR) - 70 mm/hour). The blood picture showed fragmented red blood cells, schistocytes, thrombocytopenia, predominant neutrophils with more band forms, and these findings were compatible with bacterial infection and hemolytic anemia. The direct Coombs test was weakly positive. Renal function had deteriorated (blood urea - 48 mg/dl, serum creatinine - 1.1 mg/dL, K - 5.1 mmol/l, Na - 142/mmol/l). Urine culture showed no growth. The urine full report showed proteinuria, moderate presence of red blood cells, and a field full of pus cells. Liver function was elevated (alanine transaminase (ALT) - 84 U/L, aspartate aminotransferase (AST) - 120 U/L, serum bilirubin - 3 mg/dl with the predominant indirect fraction - 2 mg/dl). The coagulation profile was normal and d-dimers were negative. Testing for other primary aetiologies for HUS such as HIV antibodies, influenza antibodies, serum complements, antinuclear antibodies, and stool cultures for bacteria was negative. The diagnosis of HUS secondary to Streptococcus pneumoniae was made based on the Canadian Paediatric Society Streptococcus pneumoniae-associated hemolytic uremic syndrome case definitions.

He was managed with renal replacement therapy and platelet and blood transfusions for five days. Antihypertensives were required temporarily to manage high blood pressure. His clinical, biochemical, and hematological parameters gradually recovered over the following 14 days. Intravenous ceftriaxone was given for 14 days and a renal dose of vancomycin was given for seven days. CXR and all other investigations had been normal at discharge. He was reviewed in the pediatric clinic two weeks after discharge and was clinically and biochemically normal with no residual complications.

## Discussion

Ninety percent of childhood HUS is caused by STEC (Shiga-toxin producing E. coli), and Streptococcus (S.) pneumoniae as the primary etiology of HUS is unusual [8]. The incidence of SpHUS following an invasive S. pneumoniae infection is recognized to be less than 5% in all children with HUS. Before the introduction of the pneumococcal vaccine, the prominent serotypes of SpHUS were 14, 6B, 9V, 19, 3, 8, and 23F but after the introduction of the seven-valent pneumococcal protein conjugate vaccine (Prevnar) in 2000, there was a shift of SpHUS-associated strains that were not protected by the vaccine. The newly developed 13-valent vaccine (Prevnar 13) and 23-valent vaccine (Pneumovax 23) are more promising against most strains of SpHUS [9]. As invasive pneumococcal infections occur frequently in children under two years, they have a higher risk of HUS, and SpHUS is usually subordinated with pneumonia, pleural effusion, or empyema in the majority of patients [9]. Our child was not vaccinated with the pneumococcal vaccine since the pneumococcal vaccine is not offered in the Expanded Program in Immunization (EPI) schedule in Sri Lanka. Meningitis is the second most common complication associated with Streptococcus pneumoniae infection and carries a high mortality rate.

The pathogenesis of HUS following STEC differs from SpHUS. Neuraminidase produced by Streptococcus pneumoniae mediates HUS in SpHUS by promoting the binding of T-antigen-specific antibodies on to the T antigen present on the surface of red blood cells, glomerular endothelial cells, and platelets. The T antigen, which is also known as the Thomsen Friedenreich antigen, is present as a normal structure on the surface of erythrocytes, platelets, and renal capillary endothelial cells, and N-acetylneuraminic acid present on the same surfaces prevent the T antigen being exposed to circulating T antigen-specific antibodies. Once neuraminidase cleaves neuraminic acid, circulating anti-T immunoglobulin M (IgM) antibodies binds increasingly to the T antigen, triggering an immune-mediated kidney injury and thrombocytopenia. Microangiopathy-mediated hemolysis and increased clearance of T-antigen-activated red cells lead to anemia. Patients with Streptococcal pneumoniae-associated HUS have low levels of circulating anti-T antigen IgM [7,10-11]. As this activated T antigen is also present on hepatocytes and in the choroid plexus in the brain, it might cause hepatic dysfunction and meningitis [12-13].

There were several similar reports where HUS-associated streptococci pneumoniae had been reported following focal invasive pneumococcal infections such as pneumonia with empyema, meningitis, sepsis, otitis media, and, mastoiditis [2,10,13]. This child had both pneumonia and meningitis before the course was further complicated by HUS.

Early detection of S pneumoniae-associated HUS is vital as unwashed plasma or plasma-containing blood products may worsen the condition and many healthy individuals have anti-T IgM in their serum. Fluorescein-labeled peanut agglutinin (Arachis hypogaea) confirms the presence of the T antigen on tested cells or tissues, but it is not routinely done. Instead, the direct Coombs test can identify autoantibodies binding to red blood cells [10,13]. The reported child had a weakly positive Coombs test. Also, mild liver function abnormalities had been found in most patients including in our patient [14]. There is no consistent case definition for S, pneumoniae-associated HUS. Both the Canadian Paediatric Society and the Centers for Disease Control and Prevention currently have proposed case definitions for post-diarrheal HUS [15] but not for S pneumoniae-associated HUS alone. The Canadian Paediatric Surveillance Program case definition [16] is outlined in Table 1. The diagnosis can be made based on findings consistent with the microangiopathic hemolytic anemia, biochemical, renal, and hematological abnormalities noted in Table 1. In circumstances in which the diagnosis or etiology is unclear, a renal biopsy could be considered.

**Table 1:** Canadian Paediatric Society Streptococcus pneumoniae-associated hemolytic uremic syndrome case definitions

Children under the age of 16 years demonstrating the following (need not be present simultaneously)
1) Evidence of invasive S pneumoniae infection (blood or another normally sterile biological fluid: cerebrospinal, pericardial, articular, peritoneal, pleural), excluding middle ear, sinus, and tracheal aspirates
2) Both renal and hematological organ failures defined as a) Acute renal impairment with serum creatinine: ▪ >50 μmol/L if younger than five years old ▪ >60 μmol/L if five to nine years old ▪ >90 μmol/L if 10 to 13 years old ▪ >110 μmol/L if older than 13 years old, b) Microangiopathic hemolytic anemia (hemoglobin <100g/L with fragmented red cells), c) Thrombocytopenia (<150,000x109/L) in the absence of septicemia, malignant hypertension, chronic uremia, collagen, or vascular disorders
3) No chronic conditions that are causative for the renal and hematological abnormalities seen. Other organ failures may occur
Definite case: Evidence of thrombotic microangiopathy on renal biopsy or autopsy
Possible case: The distinction between pneumococcal sepsis with secondary organ failures and S pneumoniae -associated hemolytic uremic syndrome will be determined through a Delphi process

The American Academy of Paediatrics recommends vancomycin and extended-spectrum cephalosporins for patients with invasive pneumococcal infections due to the high incidence of antibiotic resistance in the community [17]. The reported child showed a rapid response to combined therapy with vancomycin and ceftriaxone after initial poor response to intravenous cefotaxime alone. Although blood culture and cerebrospinal fluid (CSF) culture revealed resistance to penicillin and sensitivity to cefotaxime, the invasive nature of Streptococcus pneumoniae in this child led to a lack of response to cefotaxime. While receiving treatment for streptococcus-associated septicemia and meningitis, he developed HUS. The management of Streptococcus pneumoniae-associated HUS is supportive. Fresh frozen plasma should be avoided in children with no bleeding manifestations. If red cells and platelets are to be transfused, they need to be washed, as anti-TF IgM formation is central in the pathogenesis of SpHUS.

Hemodialysis is indicated for children with acute kidney injury. Our child had transient renal impairment which needed short-term renal replacement therapy and control of hypertension for five days. It is also important to avoid unwashed blood products to prime the dialysis system. The prevention of SpHUS is mainly by vaccination of children with the 13-valent pneumococcal conjugate vaccine (PCV13) or the 23-valent pneumococcal polysaccharide vaccines. Although dramatic reductions of invasive pneumococcal infections were seen with increased vaccination coverage, the emergence of invasive infections caused by strains that are not covered by the pneumococcal vaccine remains a risk [18].

## Conclusions

Streptococcus pneumoniae is an unusual cause of HUS and is associated with worse outcomes as compared to STEC-mediated HUS. Early diagnosis and treatment are crucial for the prevention of long-term sequelae. Complications such as meningitis and HUS have to be suspected early in children diagnosed with invasive Streptococcus pneumoniae infections and in those who do not dramatically respond to conventional treatment.
